# Why is announcement training more effective than conversation training for introducing HPV vaccination? A theory-based investigation

**DOI:** 10.1186/s13012-018-0743-8

**Published:** 2018-04-19

**Authors:** Teri L. Malo, Megan E. Hall, Noel T. Brewer, Christine R. Lathren, Melissa B. Gilkey

**Affiliations:** 1 Lineberger Comprehensive Cancer Center, University of North Carolina, CB7293, Chapel Hill, NC 27599 USA; 2 Department of Health Behavior, Gillings School of Global Pubic Health, University of North Carolina, CB7440, Chapel Hill, NC 27599 USA; 3 Division of Pharmaceutical Outcomes and Policy, Eshelman School of Pharmacy, University of North Carolina, CB7355, Chapel Hill, NC 27599 USA; 4 Program on Aging, Disability, and Long-Term Care, Cecil G. Sheps Center for Health Services Research, University of North Carolina, CB7590, Chapel Hill, NC 27599 USA

**Keywords:** Adolescent health, Cancer, Health communication, Healthcare providers, Human papillomavirus vaccines, Process assessment

## Abstract

**Background:**

Improving healthcare providers’ communication about HPV vaccination is critical to increasing uptake. We previously demonstrated that training providers to use presumptive announcements to introduce HPV vaccination improved uptake, whereas training them to use participatory conversations had no effect. To understand how communication training changed provider perceptions and communication practices, we evaluated intermediate outcomes and process measures from our randomized clinical trial, with a particular focus on identifying mechanisms that might explain the announcement training’s impact.

**Methods:**

In 2015, a physician educator delivered 1-h in-clinic HPV vaccination recommendation trainings at 20 primary care clinics in North Carolina serving 11,578 patients age 11 or 12. Clinics were randomized to receive training to use “announcements” that presume parents are ready to vaccinate or “conversations” that invite dialog about vaccination. Training participants were 83 HPV vaccine providers. Pre- and post-training surveys assessed constructs from the theory of planned behavior (TPB), including providers’ attitudes and subjective norms about HPV vaccination and their perceived behavioral control to recommend HPV vaccination. Surveys also assessed providers’ perceptions of the announcement and conversation communication strategies.

**Results:**

Both trainings improved TPB-related constructs, including providers’ positive attitudes toward HPV vaccination, subjective norms, and perceived behavioral control to recommend the vaccine (all *p* < .001, Cohen’s *d* = .62–.90). Furthermore, in both trainings, the amount of time providers reported needing to discuss HPV vaccination with parents decreased from pre-training to 1-month follow-up (mean = 3.8 vs. 3.2 min, *p* = .01, *d* = .28). However, announcement trainings outperformed conversation trainings on other measures. For example, providers who received announcement training more often reported that the communication strategy saved them time, was easy to use, helped them promote HPV vaccination as routine care, and increased HPV vaccination coverage in their clinics (all *p* < .05; *d* = .44–.60).

**Conclusions:**

Both announcement and conversation trainings improved providers’ HPV vaccine-related perceptions. However, providers viewed announcements as easier to use and more effective, which may help to explain the success of this training approach. Future provider communication interventions should consider implementation outcomes, including acceptability, alongside more traditional TPB constructs.

**Trial registration:**

clinicaltrials.gov, NCT02377843. Registered on February 27, 2015.

**Electronic supplementary material:**

The online version of this article (10.1186/s13012-018-0743-8) contains supplementary material, which is available to authorized users.

## Background

Human papillomavirus (HPV) vaccination can greatly reduce morbidity and mortality related to genital warts and HPV-associated cancers [1, 2]. National recommendations call for routine vaccination for females and males age 11 or 12 [3]. The vaccine may be most effective when delivered at this age because younger adolescents have a higher immune response [4] and a low likelihood of prior exposure to HPV. Many countries, including the United States of America (USA), have moved to a two-dose schedule for younger adolescents [5]. However, low uptake in the USA has undermined the vaccine’s promise. Only about half of US adolescents have received even one dose of HPV vaccine by age 13 [6].

Healthcare providers’ recommendations are a powerful motivator of HPV vaccine uptake, yet recommendations are often absent or of low quality [7]. Based on national practice recommendations and research findings, providers should routinely recommend same-day HPV vaccination for adolescents of both sexes at age 11 or 12 [7, 8]. However, a national survey we conducted with 776 US pediatricians and family physicians found that many physicians (59%) recommended HPV vaccine using a risk-based approach (i.e., recommended HPV vaccination preferentially for those they perceived to be at higher risk for HPV infection), and about half (49%) recommended something other than same-day vaccination [7]. An appreciable minority of physicians did not strongly recommend HPV vaccine (27%), or they recommended vaccination after age 12 or not at all for females (26%) or males (39%). Efforts are needed to increase recommendation frequency and quality. These efforts should consider facilitators of and barriers to provider communication, including providers’ perceptions of time constraints during the clinical encounter [9–11].

Until recently, a key barrier to improving communication about HPV vaccination was a lack of data about how to best introduce the topic of adolescent vaccination. In their observational research to understand how providers communicate with vaccine-hesitant parents about early childhood vaccines, Opel and colleagues found that most providers used one of two approaches to initiate their vaccine recommendations: a presumptive approach (which we refer to as an “announcement”) or a participatory approach (or “conversation”) [12]. In the case of announcements, providers initiated their vaccine recommendations using statements that presumed parents were ready to vaccinate (e.g., “Your child is due for three vaccines”). Providers using a participatory approach instead engaged parents in dialogue [13] (e.g., “What do you want to about the vaccines today?”).

To evaluate these two communication approaches using a more rigorous study design [12, 14, 15], we conducted a randomized clinical trial of 30 primary care clinics in North Carolina to assess the impact of announcement and conversation communication training on HPV vaccination coverage. We developed the trainings based in part on the theory of planned behavior (TPB), a theory that has shown promise in predicting pediatricians’ HPV vaccine recommendation behaviors [16]. This theory posits that one’s attitude toward a behavior, perceptions of subjective norms about a behavior, and beliefs about one’s capability of performing a behavior and its impact (i.e., perceived behavioral control) increase intentions to act (e.g., intentions to recommend HPV vaccine), which, in turn, leads to the behavior (e.g., HPV vaccine recommendation) [17]. Thus, we operationalized these TPB constructs as providers’ positive attitudes toward, perceived subjective norms about, and perceived behavioral control to recommend HPV vaccination. We anticipated that changes in these constructs would lead to changes in providers’ communication practices for recommending HPV vaccination. Formative research to inform the trainings included national surveys of US primary care physicians [7, 9] and parents of adolescents [18]; other published findings including those suggesting the utility of the TPB for understanding patient-provider communication about HPV vaccine [16]; feedback from an expert panel of pediatricians, family physicians, other vaccine providers, and researchers; and pilot testing with two clinics in North Carolina.

Among 11- to 12-year-old patients, we found that training providers to make presumptive announcements about HPV vaccine increased coverage by 5%, whereas training providers to start participatory conversations did not result in statistically significant improvements in coverage [19]. An important next step is to understand the mechanisms by which communication training worked to improve vaccine delivery by exploring possible mechanisms for the success of the announcement arm. Thus, the aims of the present study were to evaluate the two trainings’ impact, both overall and by trial arm, on (1) intermediate outcomes, including providers’ TPB-related perceptions and self-reported recommendation practices and (2) process measures such as acceptability. By identifying specific ways in which announcement training outperformed conversation training, this study seeks to inform the national dissemination of our intervention protocol.

## Methods

We conducted a randomized clinical trial to evaluate the impact of provider trainings on HPV vaccine communication (NCT02377843). We randomly assigned clinics to one of two intervention arms (announcement training or conversation training) or to a waitlist control arm, and then we recruited the clinics to meet our quota. This article focuses on the 20 clinics that received the communication trainings; we did not provide training to clinics in the no-intervention control arm during the follow-up period. We described our methods previously [19] and thus present them briefly below.

### Participants and setting

Clinics were eligible to enroll in the trial if they specialized in pediatrics or family medicine; had 100 or more patients age 11 or 12 attributed to the clinic in the North Carolina Immunization Registry; were located within a 2-h drive of Chapel Hill, North Carolina; and had at least one pediatric or family medicine physician who provided HPV vaccine to 11- or 12-year-olds. We identified 150 eligible clinics that we randomized. We recruited to meet the trial quota of 10 enrolled clinics per arm (30 clinics total). Intervention clinics received up to $800, and their providers received up to one continuing medical education credit for attending the training. Because vaccine delivery happens in the context of a health care team, both prescribing clinicians (e.g., physicians, physician assistants, and nurse practitioners) and non-prescribing clinicians (e.g., nurses) participated in trainings and completed related surveys. Given our study’s special focus on prescribing, vaccine-prescribing clinicians also completed a follow-up survey for which they received $100. Providers practicing at the clinics consented to be in the trial before the training began. The University of North Carolina Institutional Review Board approved the trial protocol.

### Intervention

From May to August 2015, a physician educator delivered the 1-h trainings to vaccine-prescribing clinicians and other staff at intervention clinics. The physician educator led the four-part training using a standardized script and PowerPoint slide set. The first section, a review of HPV vaccination research and the reason for focusing on younger adolescents, was designed to affect attitudes (e.g., that their recommendation increases HPV vaccination) and subjective norms (e.g., that parents think HPV vaccination is important). The second and third sections were designed to bolster providers’ perceived behavioral control (e.g., knowing how to recommend HPV vaccine in a way that leads to vaccination). In the second section, the physician educator taught participants a strategy for delivering effective HPV vaccine recommendations by starting with either an announcement or conversation strategy and then, as needed, addressing parent questions using the EASE approach, and recommending the vaccine. The EASE approach to addressing questions was to *elicit* the parent’s main concern, *acknowledge* the concern without judgment, *share* a commitment to vaccination and the child’s health, and *explain* what the science says. Trainings were identical except for the recommendation strategy taught during this section. In the third section, participants developed a brief script for the first step of the strategy and practiced it with a colleague. In the last section, participants discussed how they would apply the recommendation strategy to their clinical practice.

### Measures

Vaccine-prescribing clinicians completed three written surveys over the course of the study: a *pre-training survey* immediately prior to the start of the training, a *post-training survey* at the end of the training, and a *follow-up survey* by mail or online after practicing the recommendation strategy with at least five patients. Other providers (non-vaccine prescribing clinicians) completed only the pre- and post-training surveys. We developed items using validated measures [7] when possible. We created new items using a rigorous process that included pretesting with four physicians to assess item comprehension. Surveys are available at http://www.unc.edu/~ntbrewer/hpv.htm, in Additional file 1: Table S1, and in Additional file 2.

#### HPV vaccine recommendation behaviors

Pre-training and 1-month follow-up surveys assessed self-reported recommendation behavior. One item assessed use of the announcement approach to introducing HPV vaccination: “Some clinicians first talk about adolescent vaccines by *announcing* the child is due for meningitis, HPV, and Tdap vaccines, and then saying, ‘We’ll give those at the end of the visit.’ How often did you use this approach when talking about HPV vaccination in the last two weeks?” Another item assessed use of the conversation approach to introducing HPV vaccination: “Some clinicians first talk about adolescent vaccines by *starting a conversation about* the health benefits of meningitis, HPV, and Tdap vaccines, and then asking, ‘What questions do you have?’ How often did you use this approach when talking about HPV vaccination in the last two weeks?” Both items examined a concurrent recommendation for all three vaccines in the routine adolescent immunization schedule, an approach included in the trainings and advocated by the Centers for Disease Control and Prevention [20]. The 5-point response scale ranged from never to always.

Pre-training and 1-month follow-up surveys assessed recommendation quality. The six items included four items from a validated index about recommendation timeliness, urgency, consistency, and strength of endorsement [7], as well as two items from a recent systematic review about emphasizing cancer prevention and endorsing HPV vaccination as part of routine care [8]. The 5-point response scale ranged from strongly disagree to strongly agree. We created a recommendation quality index by assigning 1 point for agree or strongly agree responses and 0 points for other responses (we reverse coded the consistency variable), then summing responses to create a composite score (range 0–6).

#### Time spent discussing HPV vaccination with patients

Pre-training and 1-month follow-up surveys assessed providers’ perceptions of the number of minutes and seconds it usually takes to talk about HPV, Tdap, and meningococcal vaccines.

#### TPB constructs

Pre- and post-training surveys included six items that assessed TPB-related constructs so as to understand the impact of our training on providers’ HPV vaccine-related perceptions; 1-month follow-up surveys also assessed three of these items. These items assessed providers’ HPV vaccine attitudes (two items), subjective norms about HPV vaccination (two items), and self-efficacy to recommend HPV vaccination (which is an aspect of perceived behavioral control, two items). We evaluated one additional construct, behavioral intentions, at post-training and 1-month follow-up with a single item that assessed intentions to use the recommendation strategy taught in the training. For all TPB items, the 5-point response scale ranged from strongly disagree to strongly agree.

#### Perceptions of the communication strategy

To evaluate the acceptability of the communication trainings, post-training and 1-month follow-up surveys assessed perceptions of the recommendation strategy taught in the training (seven items). Items assessed providers’ perceptions of the extent to which using the communication strategy is easy to do, helps them make HPV vaccination part of routine adolescent care, helps them address parents’ concerns, helps them emphasize HPV vaccination as a way to prevent cancer, saves them time, and increases HPV vaccination in their practice. The survey also asked providers whether they thought that parent satisfaction with clinic visits increased or decreased as a result of using the communication strategy. The 5-point response scale ranged from strongly disagree to strongly agree, or decreased a lot to increased a lot.

### Data analysis

Our analysis sought to first identify how communication training impacted intermediate outcomes and process measures for the two trial arms combined, and then compared the announcement training to the conversation training. We used paired *t*-tests to compare survey responses across two time points (pre- vs. post-training, or post-training vs. 1-month follow-up) and independent samples *t*-tests to compare responses by trial arm. We also examined the interaction of trial arm and time (i.e., pre-training and follow-up) using 2 × 2 analysis of variance (ANOVA) for mixed designs and report these when statistically significant. Because our trial primarily focused on vaccine-prescribing clinicians, our analyses also focus on these providers except where noted. We conducted analyses in SAS v. 9.4 (Cary, NC) using two-tailed tests and a critical alpha of .05.

## Results

We trained 83 vaccine-prescribing and 59 non-vaccine prescribing clinicians practicing at 20 clinics serving 11,578 adolescents age 11 or 12 and 24,069 adolescents ages 13 through 17. We received pre-training, post-training, and 1-month follow-up surveys from 100% of vaccine-prescribing clinicians. Most vaccine prescribers were pediatricians (65%), were female (69%), and had been practicing for at least 10 years (66%) (Table 1). The largest proportion of providers reported seeing 10–19 patients ages 11–17 years per week (61%). Roughly half of providers reported that about half or most of their patient volume is comprised of 11- or 12-year-olds (49%). Trial arms did not differ on these sample characteristics (all *p* > .05).

**Table 1:** Sample characteristics of vaccine-prescribing clinicians by trial arm (*n* = 83)

	Announcement arm (*n* = 36) *n* (%)	Conversation arm (*n* = 47) *n* (%)	*p*
Specialty			.08
Pediatrician	22 (61)	32 (68)	
Family physician	2 (6)	3 (6)	
Physician assistant	3 (8)	9 (19)	
Nurse practitioner	9 (25)	3 (6)	
Sex			.64
Male	10 (28)	16 (34)	
Female	26 (72)	31 (66)	
Years in practice			.21
0–4	11 (31)	5 (11)	
5–9	4 (11)	8 (17)	
10–14	4 (11)	10 (21)	
15–19	6 (17)	9 (19)	
≥20	11 (31)	15 (32)	
No. of 11–17-year-old patients/week			.38
1–9	8 (22)	5 (11)	
10–19	20 (56)	31 (66)	
≥ 20	8 (22)	11 (23)	
Portion of patient volume that is ages 11–12			.19
Some	19 (53)	23 (49)	
About half	13 (36)	23 (49)	
Most	4 (11)	1 (2)	

*Note*. Analyses for the items in the remaining tables and figures indicated no statistically significant difference between trial arms at baseline

### HPV vaccine recommendation behaviors

**Fig. 1:**
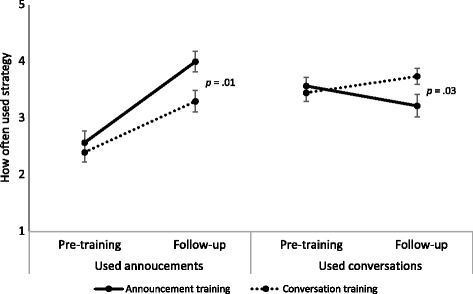
Frequency of using announcement and conversation recommendation strategies (*n* = 83)

Providers shifted their communication style to match the style in the training they attended. Before the training, providers reported using conversations more often than announcements (mean [*M*] = 3.5 vs. 2.5, *p* < .001, Cohen’s *d* = .68), a finding that did not differ by trial arm (Fig. 1). A month later, providers who received the announcement training reported a higher frequency of using announcements compared to providers in the conversation training (*M* = 4.0 vs. 3.3, *p* = .01, *d* = .58). Similarly, providers who received the conversation training reported a higher frequency of using conversations at 1-month follow-up compared to providers who received the announcement training (*M* = 3.7 vs. 3.2, *p* = .03, *d* = .49). For use of conversations, the interaction between time and trial arm was statistically significant (*p* = .03) due to rising use of conversations in the conversation training arm and falling use in the other arm.

Quality of recommendation practices increased as well for both trial arms combined. The recommendation quality index increased from an average of 4.9 (standard deviation [*SD*] = 1.2, range 0–6) at pre-training to 5.3 (*SD* = 0.7, range 2–6) at 1-month follow-up (*p* = .001, *d* = .36). In exploratory analyses of individual items in the recommendation quality index, we found increases from pre-training to 1-month follow-up in four measures: vaccine recommendation timeliness (*p* < .001, *d* = .39), urgency (*p* < .001, *d* = .49), and strength of endorsement (*p* < .001, *d* = .50); and promoting HPV vaccination as part of routine care (*p* = .02, *d* = .27) (Table 2). Emphasis on cancer prevention in discussions and recommendation consistency did not change. While trial arms did not differ on recommendation quality measures by 1-month follow-up (all *p* > .05), change in recommendation quality differed for the two trial arms (*p* < .05 for interaction). For conversation training participants, the index increased from an average of 4.7 (*SD* = 1.4, range 0–6) at pre-training to 5.3 (*SD* = 0.8, range 2–6) at 1-month follow-up (*p* = .001, *d* = .49). For announcement training participants, there was a not statistically significant increase in quality of recommendation practices index, from an average of 5.2 (*SD* = 0.8, range 3–6) at pre-training to 5.3 (*SD* = 0.6, range 4–6) at 1-month follow-up (*p* = .38).

**Table 2:** HPV vaccine recommendation quality (*n* = 83)

	Pre-training *M* (*SD*)	One-month follow-up *M* (*SD*)
Recommendation quality index	4.9 (1.2)	5.3 (0.7)*
Recommendation quality items		
I start routinely recommending HPV vaccine when patients turn 11 or 12. (*timeliness*)	4.3 (0.8)	4.6 (0.5)*
I recommend HPV vaccine more often for adolescents at higher risk for getting HPV. (*consistency*, reverse coded in quality index)	3.0 (1.3)	3.0 (1.3)
When I recommend HPV vaccine, I say it is very important. (*strength of endorsement*)	4.1 (0.8)	4.4 (0.6)*
When I recommend HPV vaccine, I recommend getting it that day. (*urgency*)	4.2 (0.7)	4.5 (0.6)*
I promote HPV vaccination as part of routine adolescent care. (*routine care*)	4.6 (0.6)	4.7 (0.5)*
When I recommend HPV vaccine, I emphasize that it can prevent cancer. (*cancer prevention*)	4.7 (0.5)	4.7 (0.5)

*Note*. The 5-point response scale ranged from strongly disagree (coded as 1) to strongly agree (5). Table not stratified by trial arm because they largely did not differ

*p < .05

### Time spent discussing HPV vaccination with patients

**Fig. 2:**
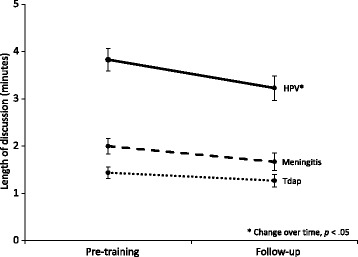
Time it takes to discuss adolescent vaccines (*n* = 83)

The amount of time providers said they spent discussing HPV vaccine decreased from pre-training to 1-month follow-up (*M* = 3.8 vs. 3.2 min, *p* = .01, *d* = .28) (Fig. 2). Time spent discussing the other vaccines did not decrease for meningitis (*M* = 2.0 vs. 1.7 min, *p* = .07) or for Tdap (*M* = 1.4 vs. 1.3 min, *p* = .26). Providers in the announcement arm, compared to the conversation arm, had higher perceptions at 1-month follow-up that using the strategy saved them time (*p* = .01, *d* = .58) (Table 3).

**Table 3:** Perceptions of the communication strategy at post-training and 1-month follow-up, by trial arm (*n* = 83)

	Announcement		Conversation	
	Post-training *M* (*SD*)	One-month Follow-up *M* (*SD*)	Post-training *M* (*SD*)	One-month Follow-up *M* (*SD*)
Using this communication strategy [will be/is] easy for me to do.	4.7 (0.5)	4.6 (0.5)	4.4 (0.7)*	4.4 (0.5)
Using this communication strategy [will help me to promote/helps me make] HPV vaccination [as] part of routine adolescent care.	4.8 (0.4)	4.6 (0.5)	4.6 (0.7)*	4.3 (0.7)*
Using this communication strategy [will help/helps] me address parents’ HPV vaccine concerns.	4.8 (0.4)	4.3 (0.8)	4.4 (0.7)*	4.4 (0.7)
Using this communication strategy will help me emphasize HPV vaccine as a way to prevent cancer.	4.9 (0.4)	–	4.6 (0.7)*	–
Using this communication strategy saves me time.	–	4.2 (0.8)	–	3.7 (0.8)*
Using this communication strategy increases HPV vaccination in my clinic or practice.	–	4.4 (0.7)	–	3.9 (0.8)*
As a result of using this communication strategy, do you think parent satisfaction with clinic visits…^a^	–	3.8 (0.6)	–	3.6 (0.6)

*Note*. Unless indicated otherwise, the response scale had 5 points that ranged from strongly disagree (coded as 1) to strongly agree (5). We did not compare changes from post-training to 1-month follow-up within trial arms due to differences in item wording at each time point

*p < .05 between trial arms at a given time point (between subjects)

^a^Response scale is 5 points, ranging from decreased a lot (coded as 1) to increased a lot (5)

– Item not assessed at this time point

### TPB constructs

**Fig. 3:**
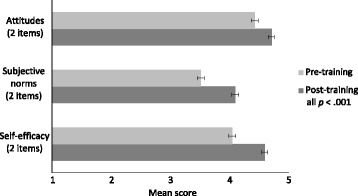
Increases in HPV vaccination attitudes, subjective norms, and perceived behavioral control (*n* = 83)

Providers had increases in positive attitudes toward HPV vaccination from pre- to post-training (*M* = 4.4 vs. 4.7, *p* < .001, *d* = .62) (Fig. 3). They also had increases in subjective norms (*M* = 3.5 vs. 4.1, *p* < .001, *d* = .90) and perceived behavioral control to recommend the vaccine (*M* = 4.1 vs. 4.6, *p* < .001, *d* = .89). Of the measures assessed at 1-month follow-up, all remained higher than at pre-training (all *p* < .01) (Table 4). Trial arms did not differ for individual HPV vaccine perception items at post-training (all *p* > .05). By 1-month follow-up, trial arms differed for one perceived behavioral control item. Providers who received announcement training, compared to conversation training, reported greater levels of agreement that they felt confident addressing parents’ concerns when discussing HPV vaccine (*M* = 4.8 vs. 4.4, *p* < .01, *d* = .73). Providers’ intentions to use the recommendation strategy taught in the training did not change from post-training to 1-month follow-up nor did their intentions differ by training received (announcement vs. conversation). Among non-vaccine prescribers, participation in the training also was generally associated with pre- to post-training increases in attitudes, subjective norms, and perceived behavioral control (most *p* < .05) (Table 5). Among non-vaccine prescribers, perceptions that most parents think HPV vaccination is important for their 11- or 12-year-olds did not change (*p* = .17).

**Table 4:** Theory of planned behavior constructs among vaccine-prescribing clinicians (*n* = 83)

	Announcement arm			Conversation arm		
	Pre-training *M* (*SD*)	Post-training *M* (*SD*)	One-month follow-up *M* (*SD*)	Pre-training *M* (*SD*)	Post-training *M* (*SD*)	One-month follow-up *M* (*SD*)
Attitudes						
HPV vaccine is effective.	4.3 (0.5)	4.8 (0.4)*	–	4.6 (0.7)	4.7 (0.6)	–
A clinician’s recommendation greatly increases HPV vaccination.	4.5 (0.7)	4.7 (0.5)*	–	4.3 (0.7)	4.7 (0.6)*	–
Subjective norms						
HPV vaccine coverage is much lower than Tdap vaccine coverage in North Carolina.	4.3 (0.8)	4.7 (0.5)*	–	4.3 (0.7)	4.5 (0.7)	–
Most parents think HPV vaccination is important for their 11- or 12-year-olds.	2.7 (0.8)	3.6 (1.0)*	3.3 (1.0)*	2.7 (0.9)	3.7 (0.9)*	3.1 (0.9)*
Perceived behavioral control^a^						
When discussing HPV vaccine, I feel confident addressing parents’ concerns.	4.4 (0.6)	4.7 (0.5)*	4.8 (0.4)*	4.2 (0.7)	4.6 (0.5)*	4.4 (0.5)^†^
I know how to recommend HPV vaccine in a way that leads to vaccination.	3.9 (0.6)	4.6 (0.5)*	4.5 (0.6)*	3.8 (0.7)	4.6 (0.5)*	4.3 (0.5)*
Behavioral intentions						
I plan to [use/routinely use] this communication strategy to recommend HPV vaccine for my adolescent patients.	–	4.6 (1.0)	4.6 (0.8)	–	4.5 (1.0)	4.4 (0.7)

*Note*. The 5-point response scale ranged from strongly disagree (coded as 1) to strongly agree (5)

^a^The items assessed self-efficacy, which is one component of perceived behavioral control

*p < .05 compared to pre-training, within the trial arm

^†^*p* < .01 compared to the announcement arm

– Item not assessed at this time point

**Table 5:** Theory of planned behavior constructs among non-vaccine prescribing clinicians (*n* = 59)

	Pre-training *M* (*SD*)	Post-training *M* (*SD*)
Attitudes		
HPV vaccine is effective.	3.9 (0.9)	4.5 (0.6)*
A clinician’s recommendation greatly increases HPV vaccination.	4.2 (0.7)	4.5 (0.7)*
Subjective norms		
HPV vaccine coverage is much lower than Tdap vaccine coverage in North Carolina.	3.8 (1.0)	4.2 (0.8)*
Most parents think HPV vaccination is important for their 11 or 12 year olds.	3.1 (0.9)	3.3 (0.9)
Perceived behavioral control^a^		
When discussing HPV vaccine, I feel confident addressing parents’ concerns.	3.8 (0.6)	4.3 (0.6)*
I know how to recommend HPV vaccine in a way that leads to vaccination.	3.7 (0.6)^b^	4.3 (0.6)*

*Note*. The 5-point response scale ranged from strongly disagree (coded as 1) to strongly agree (5). Table not stratified by trial arm because they did not differ

^a^The items assessed self-efficacy, which is one component of perceived behavioral control

^b^Missing data for 21 non-vaccine prescribing clinicians at pre-training due to skip pattern

*p < .05

### Perceptions of the communication strategy

Announcement training, compared to conversation training, elicited more favorable post-training perceptions that using the strategy would be easy to do (*p* = .02, *d* = .52), help them promote HPV vaccination as part of routine adolescent care (*p* = .04, *d* = .44), help them address parents’ HPV vaccine concerns (*p* < .01, *d* = .63), and help them emphasize HPV vaccine as a way to prevent cancer (*p* = .02, *d* = .47) (Table 3). Differences between arms were sustained at 1-month follow-up only with regard to perceptions that using the strategy helps the provider make HPV vaccination part of routine adolescent care (*p* < .05, *d* = .42). Providers in the announcement arm also had more favorable perceptions at 1-month follow-up that using the strategy increased HPV vaccination in their clinic or practice (*p* = .01, *d* = .60).

## Discussion

A strong provider recommendation can substantially increase HPV vaccine acceptance, but many recommendations are of low quality or absent [18]. After a 1-h in-clinic training, providers in our trial reported delivering recommendations that were stronger, timelier, more urgent, and more consistent than at pre-training. Providers also reported spending less time discussing HPV vaccination with patients following the training, suggesting that the communication strategy learned during the training may have helped save them time. Notably, results suggest both communication strategies largely were well-received by the providers, but the announcement training was somewhat better received.

Following the trainings, providers reported that the time it took to recommend HPV vaccination fell by about 20%, even as it remained more time-consuming to recommend than two other adolescent vaccines. Given that providers have little extra time, it is encouraging that a brief continuing medical education activity could make them more efficient while also increasing recommendation quality. These benefits may be important points to highlight when promoting the announcement training to providers.

To the best of our knowledge, our trial is the first to show that training can increase providers’ HPV vaccine recommendation quality. Providers reported making recommendations that were timelier, stronger, more urgent, and routine after the training, but in two areas quality did not increase. Communicating HPV vaccination as cancer prevention did not increase, perhaps because it was already so common. However, the lack of change in providers’ consistency of HPV vaccine recommendation—i.e., recommending vaccine for all children regardless of perceived HPV risk—represents a concerning divergence from national recommendations for routine HPV vaccine delivery [3]. Indeed, this pitfall is common, as only 41% of physicians in a national survey reported they avoided using a risk-based approach to recommending HPV vaccine [7]. Because adolescent sexual behavior is unpredictable and underestimated [21, 22], using a risk-based recommendation approach for HPV vaccination will leave many adolescents vulnerable to infections that may lead to cancer. Our finding highlights the need for further research on ways to improve this aspect of provider communication. Modifying the training to include a stronger emphasis on the importance of and rationale for routine HPV vaccination may improve providers’ recommendation consistency.

Our training was based in part on the TPB, which suggests that attitudes, subjective norms, and perceived behavioral control will motivate behavior [17], and has been used to study patient-provider communication about HPV vaccine [16]. Our trial saw increases in attitudes, subjective norms, and perceived behavioral control, and these increases generally were sustained for variables measured at 1-month follow-up. Providers also indicated that they planned to use (assessed at post-training) and routinely used (at 1-month follow-up) the communication strategy to recommend HPV vaccine for their adolescent patients. Non-vaccine prescribing clinicians who attended our trainings also reported increases in attitudes, subjective norms, and perceived behavioral control. This finding is important because some research suggests that medical assistants, nurses, or other providers make the initial HPV vaccine recommendation for patients [23].

Comparing trial arms holds substantial interest because, as we previously reported, announcement training increased HPV vaccine coverage among young adolescents, whereas conversation training did not [19]. Trial arms showed few differences in TPB-related perceptions and self-reported recommendation practices, perhaps because the portions of trainings that were designed to affect these perceptions and practices were designed to be the same across arms. Instead, we observed differences by trial arm in perceptions of the communication strategy, with a fairly consistent pattern of providers reporting more positive perceptions of the announcement training. We speculate that providers perceived announcements as being more feasible and therefore implemented that strategy more often. It may be that providers’ positive perceptions of the announcement training, along with TPB-related constructs, were needed to drive the higher HPV vaccine coverage we observed in announcement training clinics.

Our study contributes novel findings to the HPV vaccination literature by examining the effects of training on HPV vaccine recommendation behaviors, time it takes to make these recommendations, and TPB constructs in the context of HPV vaccination. Its strengths include a 100% response rate among vaccine-prescribing physicians who attended the trainings. Limitations of this study of providers include the relatively short, 1-month follow-up period for some survey responses and immediate post-training assessment of others; it is unclear whether changes would sustain over the long term. Evaluating changes in constructs after the intervention but before providers had an opportunity to change their behavior can disentangle the impact of the intervention from subsequent behavior which might also cause changes in these constructs [24]. Our surveys were brief, assessing TPB constructs with as few as two items, in an effort to be mindful of providers’ limited time and to increase the response rate. We cognitively tested our measures with four providers, but additional validation may be warranted in future studies. Providers’ communication behaviors were self-reported and may reflect social desirability or other biases. Furthermore, we used within-arm comparisons to examine changes over time that coincided with the training. The analysis approach yields the most easily interpreted findings when comparing measures between pre- and immediate post-training, but comparisons to 1-month follow-up did not account for secular trends. We previously reported that intervention reach and dose received were similarly high among vaccine prescribers in both trial arms [19]; however, implementation outcomes and contextual factors that we did not assess may further explain our trial outcomes. While it is encouraging that intentions to use the training approach were high and did not drop over the 1-month follow-up period, the absence of a baseline assessment meant we could not compare these measures to before we taught the communication approach. We also did not assess the broader construct of intentions to recommend HPV vaccination to all eligible patients. Future studies are needed to understand how communication training affects the experience of adolescent patients and their parents; like providers, families may appreciate spending less time on routine preventive services so as to focus on more complex health issues, such as diabetes, asthma, and depression. Finally, future studies should consider collecting survey outcomes and process data from providers in the control arm. These data would offer additional insight into how the trainings acted on the TPB constructs.

### Implications for implementation science

Similar to other studies that have used the TPB to guide theory-based evaluations alongside trials [25–28], we found that the TPB was a useful tool for investigating the mechanisms for the trainings’ overall impact on providers. In our trial, we observed pre-/post-training changes in TPB constructs, which is helpful for confirming that participating providers engaged with the training. However, improvements in TPB constructs largely did not differ across trial arms and, therefore, do not seem to explain why the announcement training was effective but the conversation training was not. Instead, providers’ favorable perceptions (or acceptability) of the announcement training may have been an underlying mechanism for the announcement training’s success. The implications of our findings are that TPB constructs may not be sufficient for understanding intervention impact and likely need to be considered in the context of implementation outcomes, such as acceptability [29].

## Conclusions

Physician-led trainings increased providers’ support for HPV vaccination and the quality of their recommendations and saved them time when recommending the vaccine. Training providers to use announcements could help them make effective recommendations to their patients and, ultimately, reduce the incidence of HPV cancers. Future provider communication interventions should consider implementation outcomes, including acceptability, alongside more traditional TPB constructs.

## Additional files

Additional file 1: **Table S1.** Survey items. (DOCX 31 kb)

Additional file 2: Pre-training, post-training, and 1-month follow-up surveys. (PDF 424 kb)

## Abbreviations

ANOVA: Analysis of variance
HPV: Human papillomavirus
M: Mean
SD: Standard deviation
Tdap: Tetanus, diphtheria, and acellular pertussis
TPB: Theory of planned behavior
USA: United States of America
