# Supplementary material for: Why is announcement training more effective than conversation training for introducing HPV vaccination? A theory-based investigation
Source: Implement Sci. 2018 Apr 19;13:57. doi: 10.1186/s13012-018-0743-8 (PMC5907716; doi:10.1186/s13012-018-0743-8)
Supplement: Supplementary file 1 — Table S1. Survey items. (DOCX 31 kb) [file 13012_2018_743_MOESM1_ESM.docx]

**Additional file 1: Table S1.** Survey items.

| **Construct** | **Item** | **Response Scale^a^** | **Pre-training**  **survey** | **Post-training**  **survey** | **One-month follow-up**  **survey** |
| --- | --- | --- | --- | --- | --- |
| **HPV vaccine recommendation behaviors** |  |  |  |  |  |
| Use of communication approach in training | Some clinicians first talk about adolescent vaccines by [announcing the child is due for meningitis, HPV, and Tdap vaccines, and then saying, ‘We’ll give those at the end of the visit.’] [starting a conversation about the health benefits of meningitis, HPV, and Tdap vaccines, and then asking, ‘What questions do you have?’] How often did you use this approach when talking about HPV vaccination in the last two weeks? | Never to always | X |  | X |
|  |  |  |  |  |  |
| Timeliness | I start routinely recommending HPV vaccine when patients turn 11 or 12. | Strongly disagree to strongly agree | X |  | X |
| Consistency | I recommend HPV vaccine more often for adolescents at higher risk for getting HPV. | Strongly disagree to strongly agree | X |  | X |
| Strength of endorsement | When I recommend HPV vaccine, I say it is very important. | Strongly disagree to strongly agree | X |  | X |
| Urgency | When I recommend HPV vaccine, I recommend getting it that day. | Strongly disagree to strongly agree | X |  | X |
| Routine care | I promote HPV vaccination as part of routine adolescent care. | Strongly disagree to strongly agree | X |  | X |
| Cancer prevention | When I recommend HPV vaccine, I emphasize that it can prevent cancer. | Strongly disagree to strongly agree | X |  | X |
|  |  |  |  |  |  |
|  |  |  |  |  |  |
|  |  |  |  |  |  |
|  |  |  |  |  |  |
| **Time spent discussing vaccination with patients** |  |  |  |  |  |
| HPV vaccine | For adolescent patients, how long does it usually take you to talk about HPV vaccine? | Minutes and seconds | X |  | X |
| Tdap vaccine | For adolescent patients, how long does it usually take you to talk about Tdap vaccine? | Minutes and seconds | X |  | X |
| Meningococcal vaccine | For adolescent patients, how long does it usually take you to talk about Meningococcal vaccine? | Minutes and seconds | X |  | X |
| **Theory of Planned Behavior** |  |  |  |  |  |
| Attitudes | HPV vaccine is effective. | Strongly disagree to strongly agree | X | X |  |
| Attitudes | A clinician’s recommendation greatly increases HPV vaccination. | Strongly disagree to strongly agree | X | X |  |
| Subjective norms | HPV vaccine coverage is much lower than Tdap vaccine coverage in North Carolina. | Strongly disagree to strongly agree | X | X |  |
| Subjective norms | Most parents think HPV vaccination is important for their 11 or 12 year olds. | Strongly disagree to strongly agree | X | X | X |
| Perceived behavioral control (self-efficacy) | When discussing HPV vaccine, I feel confident addressing parents’ concerns. | Strongly disagree to strongly agree | X | X | X |
| Perceived behavioral control (self-efficacy) | I know how to recommend HPV vaccine in a way that leads to vaccination. | Strongly disagree to strongly agree | X | X | X |
| Behavioral intentions | I plan to [use/routinely use] this communication strategy to recommend HPV vaccine for my adolescent patients. | Strongly disagree to strongly agree |  | X | X |
|  |  |  |  |  |  |
| **Perceptions of the communication strategy** |  |  |  |  |  |
| Acceptability | Using this communication strategy [will be/is] easy for me to do. | Strongly disagree to strongly agree |  | X | X |
| Acceptability | Using this communication strategy [will help me to promote/helps me make] HPV vaccination [as] part of routine adolescent care. | Strongly disagree to strongly agree |  | X | X |
| Acceptability | Using this communication strategy [will help/helps] me address parents’ HPV vaccine concerns. | Strongly disagree to strongly agree |  | X | X |
| Acceptability | Using this communication strategy will help me emphasize HPV vaccine as a way to prevent cancer. | Strongly disagree to strongly agree |  | X |  |
| Acceptability | Using this communication strategy saves me time. | Strongly disagree to strongly agree |  |  | X |
| Acceptability | Using this communication strategy increases HPV vaccination in my clinic or practice. | Strongly disagree to strongly agree |  |  | X |
| Acceptability | As a result of using this communication strategy, do you think parent satisfaction with clinic visits. | Increased a lot to decreased a lot |  |  | X |

^a^ 5-point response scale for all items except those under the construct “Time spent discussing vaccination with patients.”
