# Supplementary material for: Why is announcement training more effective than conversation training for introducing HPV vaccination? A theory-based investigation
Source: Implement Sci. 2018 Apr 19;13:57. doi: 10.1186/s13012-018-0743-8 (PMC5907716; doi:10.1186/s13012-018-0743-8)
Supplement: Supplementary file 2 — Pre-training, post-training, and 1-month follow-up surveys. (PDF 424 kb) [file 13012_2018_743_MOESM2_ESM.pdf]

Pre-Training Survey

1. What is your position at this clinic or practice?
   - ☐ Pediatrician
   - ☐ Family physician
   - ☐ Other physician
   - ☐ Physician assistant
   - ☐ Nurse practitioner
   - ☐ Other (please specify):______________________________

2. How many years have you been in this or a similar position?
   (Count only years after training/residency/fellowship.)
   - ☐ <5 yrs ☐ 5-9 yrs ☐ 10-14 yrs ☐ 15-19 yrs ☐ ≥20 yrs

3. About how many 11-17 year old patients do you see in a typical week?
   - ☐ 0 ☐ 1-4 ☐ 5-9 ☐ 10-14 ☐ 15-19 ☐ ≥20

4. Of these patients, about how many are 11-12 year olds?
   - ☐ None ☐ Some ☐ About half ☐ Most ☐ All

5. Are you …
   - ☐ Male ☐ Female

Say how much you agree or disagree with the following statements about HPV vaccine for 11-12 year olds.

	Strongly disagree	Disagree	Neither agree nor disagree	Agree	Strongly agree
6. HPV vaccine coverage is much lower than Tdap vaccine coverage in North Carolina.	☐	☐	☐	☐	☐
7. HPV vaccine is effective.	☐	☐	☐	☐	☐
8. Most parents think HPV vaccination is important for their 11 or 12 year olds.	☐	☐	☐	☐	☐
9. I promote HPV vaccination as part of routine adolescent care.	☐	☐	☐	☐	☐
10. A clinician's recommendation greatly increases HPV vaccination.	☐	☐	☐	☐	☐
11. When discussing HPV vaccine, I feel confident addressing parents' concerns.	☐	☐	☐	☐	☐
12. Talking with patients and parents about HPV vaccination takes too long.	☐	☐	☐	☐	☐

13. What is your role in adolescent vaccination? Do you …
(Mark all that apply.)

- ☐ Schedule appointments
- ☐ Prescribe vaccines
- ☐ Answer parents' questions about vaccines
- ☐ Administer vaccines
- ☐ Document vaccinations
- ☐ I don't have a role in adolescent vaccination

If you prescribe vaccines or answer questions about vaccines, please continue.
Otherwise, stop here and return your survey to the facilitator.

Say how much you agree or disagree with the following statements about recommending HPV vaccine for 11-12 year olds.

	Strongly disagree	Disagree	Neither agree nor disagree	Agree	Strongly agree
14. I start routinely recommending HPV vaccine when patients turn 11 or 12.	☐	☐	☐	☐	☐
15. I know how to recommend HPV vaccine in a way that leads to vaccination.	☐	☐	☐	☐	☐
16. When I recommend HPV vaccine, I say it is very important.	☐	☐	☐	☐	☐
17. When I recommend HPV vaccine, I emphasize that it can prevent cancer.	☐	☐	☐	☐	☐
18. When I recommend HPV vaccine, I recommend getting it that day.	☐	☐	☐	☐	☐
19. I recommend HPV vaccine more often for adolescents at higher risk for getting HPV.	☐	☐	☐	☐	☐

For adolescent patients, how long does it usually take you to talk about …

20. HPV vaccine _____ minutes _____ seconds

21. Tdap vaccine _____ minutes _____ seconds

22. Meningococcal vaccine _____ minutes _____ seconds

23. Some clinicians first talk about adolescent vaccines by announcing the child is due for meningitis, HPV, and Tdap vaccines, and then saying, “We’ll give those at the end of the visit.”

How often did you use this approach when talking about HPV vaccination in the last two weeks?

☐ Never ☐ Rarely ☐ Sometimes ☐ Often ☐ Always

24. Some clinicians first talk about adolescent vaccines by starting a conversation about the health benefits of meningitis, HPV, and Tdap vaccines, and then asking, “What questions do you have?”

How often did you use this approach when talking about HPV vaccination in the last two weeks?

☐ Never ☐ Rarely ☐ Sometimes ☐ Often ☐ Always

25. At what other practices or clinics do you provide adolescent vaccines?

☐ Only at the practice or clinic where I attended the training
☐ I also provide adolescent vaccines at: ______________________ on _____ days a week
☐ I also provide adolescent vaccines at: ______________________ on _____ days a week

Thank you! Please return your completed survey to the facilitator.

Applying to Your Practice

Consider taking a first step toward routinely using this communication strategy in your practice. Whenever we think about practicing a new skill, it can be easy to forget or just "never get around to it." Making a specific plan about exactly when, where, and how we'll do something can be helpful.

My two-week plan

If within two weeks I have not used this communication strategy
to recommend HPV vaccine with five patients who are 11-12 years old,
I will use the strategy with the **next five** patients who are 11-12 years old.

☐ I agree to do this ☐ I may not do this ☐ I do not see 11-12 year old patients

While we encourage you to use the strategy with all adolescents, it is especially important to use it with 11-12 year old patients.

CME Survey

The next questions are about the **communication strategy** you learned in today's training.

Say how much you agree or disagree with the following statements about HPV vaccine for 11-12 year olds.

Using this communication strategy will…	Strongly disagree	Disagree	Neither agree nor disagree	Agree	Strongly agree
1. be easy for me to do.	☐	☐	☐	☐	☐
2. help me to promote HPV vaccination as part of routine adolescent care.	☐	☐	☐	☐	☐
3. help me emphasize HPV vaccine as a way to prevent cancer.	☐	☐	☐	☐	☐
4. help me address parents' HPV vaccine concerns.	☐	☐	☐	☐	☐

5. I plan to use this communication strategy to recommend HPV vaccine for my adolescent patients.

☐ Strongly disagree ☐ Disagree ☐ Neither agree nor disagree ☐ Agree ☐ Strongly agree

Say how much you agree or disagree with the following statements about HPV vaccine for 11-12 year olds.

	Strongly disagree	Disagree	Neither agree nor disagree	Agree	Strongly agree
6. HPV vaccine coverage is much lower than Tdap vaccine coverage in North Carolina.	☐	☐	☐	☐	☐
7. HPV vaccine is effective.	☐	☐	☐	☐	☐
8. Most parents think HPV vaccination is important for their 11 or 12 year olds.	☐	☐	☐	☐	☐
9. A clinician's recommendation greatly increases HPV vaccination.	☐	☐	☐	☐	☐
10. I know how to recommend HPV vaccine in a way that leads to vaccination.	☐	☐	☐	☐	☐
11. When discussing HPV vaccine, I feel confident addressing parents' concerns.	☐	☐	☐	☐	☐

Please tell us about your experience with this training.

	Fair	Good	Very Good	Excellent
12. Training content	☐	☐	☐	☐
13. Practical value of the training to me	☐	☐	☐	☐
14. The presenter's knowledge of the material	☐	☐	☐	☐
15. Overall satisfaction with the training	☐	☐	☐	☐

16. Would you recommend this training to a colleague?

☐ Yes ☐ No

17. List a key **strength** of this communication training:

18. List a key **weakness** of this communication training:

Thank you! Please return your completed survey to the facilitator

HPV Vaccine Communication Survey

Complete this survey after practicing the HPV vaccination communication strategy with at least five 11-12 year old patients. If you do not expect to see five 11-12 year old patients within the next month, then complete the survey after practicing with five HPV vaccine-eligible patients. **You will receive $100 for completing this survey.**

1. Which patients did you use this communication strategy with? (Mark all that apply.)
   - ☐ 11-12 year olds
   - ☐ 13-17 year olds
   - ☐ Other ages, please specify: ________________________________
   - ☐ I did not use this communication strategy in the past month **[SKIP TO QUESTION 8]**

Say how much you agree or disagree with the following statements about HPV vaccine for 11-12 year olds.

Using this communication strategy…	Strongly disagree	Disagree	Neither agree nor disagree	Agree	Strongly agree
1. is easy for me to do.	☐	☐	☐	☐	☐
2. saves me time.	☐	☐	☐	☐	☐
3. helps me make HPV vaccination part of routine adolescent care.	☐	☐	☐	☐	☐
4. helps me address parents' HPV vaccine concerns.	☐	☐	☐	☐	☐
5. increases HPV vaccination in my clinic or practice.	☐	☐	☐	☐	☐

6. As a result of using this communication strategy, do you think parent satisfaction with clinic visits …

   ☐ Decreased a lot ☐ Decreased a little ☐ Did not change ☐ Increased a little ☐ Increased a lot

7. I plan to routinely use this communication strategy to recommend HPV vaccine for my adolescent patients.

   ☐ Strongly disagree ☐ Disagree ☐ Neither agree nor disagree ☐ Agree ☐ Strongly agree

For adolescent patients, how long does it usually take you to talk about …

8. HPV vaccine _____ minutes _____ seconds
9. Tdap vaccine _____ minutes _____ seconds
10. Meningococcal vaccine _____ minutes _____ seconds

Say how much you agree or disagree with the following statements about HPV vaccine for 11-12 year olds.

	Strongly disagree	Disagree	Neither agree nor disagree	Agree	Strongly agree
11. Most parents think HPV vaccination is important for their 11 or 12 year olds.	☐	☐	☐	☐	☐
12. I start routinely recommending HPV vaccine when patients turn 11 or 12.	☐	☐	☐	☐	☐
13. I promote HPV vaccination as part of routine adolescent care.	☐	☐	☐	☐	☐
14. I know how to recommend HPV vaccine in a way that leads to vaccination.	☐	☐	☐	☐	☐
15. When I recommend HPV vaccine, I say it is very important.	☐	☐	☐	☐	☐
16. When I recommend HPV vaccine, I emphasize that it can prevent cancer.	☐	☐	☐	☐	☐
17. When I recommend HPV vaccine, I recommend getting it that day.	☐	☐	☐	☐	☐
18. I recommend HPV vaccine more often for adolescents at higher risk for getting HPV.	☐	☐	☐	☐	☐
19. When discussing HPV vaccine, I feel confident addressing parents' concerns.	☐	☐	☐	☐	☐
20. Talking with patients and parents about HPV vaccination takes too long.	☐	☐	☐	☐	☐

21. Some clinicians first talk about adolescent vaccines by <u>announcing</u> the child is due for meningitis, HPV, and Tdap vaccines, and then saying, "We'll give those at the end of the visit."

    How often did you use this approach when talking about HPV vaccination in the last two weeks?

    ☐ Never ☐ Rarely ☐ Sometimes ☐ Often ☐ Always

22. Some clinicians first talk about adolescent vaccines by <u>starting a conversation about</u> the health benefits of meningitis, HPV, and Tdap vaccines, and then asking, "What questions do you have?"

    How often did you use this approach when talking about HPV vaccination in the last two weeks?

    ☐ Never ☐ Rarely ☐ Sometimes ☐ Often ☐ Always

23. May we contact you in the future about your experience with using this communication strategy?

    ☐ Yes ☐ No

Thank you! Please return survey to Dr. Christine Lathren, UNC HB, CB7440, Chapel Hill, NC 27599
